# Genotypes and Phenotypes of MEF2C Haploinsufficiency Syndrome: New Cases and Novel Point Mutations

**DOI:** 10.3389/fped.2021.664449

**Published:** 2021-05-13

**Authors:** Lin Wan, Xinting Liu, Linyan Hu, Huimin Chen, Yulin Sun, Zhichao Li, Zhenfang Wang, Zhi Lin, Liping Zou, Guang Yang

**Affiliations:** ^1^Medical School of Chinese People's Liberation Army, Beijing, China; ^2^Department of Pediatrics, The First Medical Center, Chinese People's Liberation Army General Hospital, Beijing, China; ^3^Department of Rehabilitation, Children Hospital of Shanxi, Taiyuan, China; ^4^Department of Pediatrics, Fujian Provincial Hospital, Fuzhou, China; ^5^The Second School of Clinical Medicine, Southern Medical University, Guangzhou, China

**Keywords:** MEF2C haploinsufficiency syndrome, trio-based whole-exome sequencing, regression, MOSAIC, MEF2C gene mutations

## Abstract

**Aim:** MEF2C haploinsufficiency syndrome (MCHS) is a severe neurodevelopmental disorder. We describe the clinical phenotypes and genotypes of seven patients with MCHS to enhance the understanding of clinical manifestations and genetic alterations associated with MCHS.

**Method:** Seven patients (6 females and 1 male, aged between 2 years 5 months and 6 years) who had MEF2C mutations, and their parents underwent trio-based whole-exome sequencing; subsequently, their clinical features were assessed. A literature review of patients with MCHS was performed by searching the PubMed and Online Mendelian Inheritance in Man databases.

**Results:** Seven mutations were identified, of which six were unreported in the past; of the reported cases, five patients had *de novo* mutations but two had an undefined inheritance pattern. All patients presented delays in developmental milestones, severe intellectual disabilities and lack of speech. Six patients exhibited infantile hypotonia, five patients experienced stereotypic movements and were unable to walk, four patients exhibited poor eye contact indicative of autism and two showed poor performance. While six patients experienced seizure, five among them became seizure free after receiving anti-seizure medicine. Three patients showed a regression in their development, whereas the mothers of two patients exhibited mosaicism but were healthy without any abovementioned symptoms.

**Interpretation:** Regression was not a common phenomenon but occurred in MCHS. The prognosis of MCHS patients with epilepsy was good, but most patients can achieve a seizure-free status. Healthy people may have low-level mosaicism and carry a pathogenic MEF2C mutation.

## Introduction

Myocyte enhancer factor 2C (MEF2C) haploinsufficiency syndrome (MCHS) has been attributed to 5q14.3 microdeletions (MIM# 613443, developmental delay, stereotypic movements, epilepsy and/or cerebral malformations) (1) and was defined as any microdeletions of chromosome 5q14.3-5q15 that involved multiple genes (www.omim.org). After identifying similar clinical manifestations in patients with 5q14.3 microdeletion syndrome, the smallest common deletion regions were found to only contain the MEF2C gene (2). The MEF2C gene located in the 5q14.3 region is a member of the MEF2 transcription factor family; it regulates the number of excitatory synapses, dendrite morphology, and post-synaptic dendrite differentiation (3–5).

Haploinsufficiency of MEF2C was recognized as the pathogenic mechanism, in that patients with deletion, truncation, or missense mutation of this gene present with similar yet distinct phenotypes (6). To date, 23 patients have been reported to have pathogenic and likely pathogenic MEF2C variants or microdeletions without an involvement of other contiguous or distant genes (1, 6–14). Typical clinical characteristics include severe global developmental delay accompanied by absent speech, limited walking, seizures, and stereotypic movements (15). Here we have described a new cohort of seven patients with MEF2C point mutations, discussed their clinical features and reported six novel mutations as well as reviewed previous data of patients having pathogenic or likely pathogenic MEF2C variants or MEF2C microdeletions without the involvement of other contiguous or distant genes, which facilitated us to further clarify MCHS genotypes and phenotypes.

## Method

Seven patients with confirmed MEF2C mutations who also had developmental delay, stereotypic movements or epilepsy were enrolled from the First Medical Center of PLA General Hospital, Fujian Provincial Hospital, and Children's Hospital of Shanxi. Detailed clinical information was collected including clinical manifestations, history of epilepsy, previous development status, family history, physical examination, treatments, and findings from electroencephalogram (EEG) and magnetic resonance imaging (MRI). Genomic DNA was extracted from peripheral blood leukocytes of the patients and their parents for trio-based whole-exome sequencing (Trio-WES). In accordance with the requirements of Research Ethics Board at First Medical Center of PLA General Hospital, informed consent was obtained from the patients' parents.

Trio-WES was performed for patients and their parents. One microgram of genomic DNA extracted from blood leukocytes was used for targeted exon enrichment on a NovaSeq 6000 instrument (Illumina, San Diego, CA, USA) according to the manufacturer's recommendations for paired-end 150-bp reads. Raw data were processed as previously described. Sequence Alignment/Map format files were aligned to a human genome reference sequence (GRCh38/hg18) using BWA (Burrows–Wheeler Aligner; v0.6.7), and potential duplicate paired-end reads were removed using Picard 1.109. Indel realignment and base quality score recalibration were performed using the Genome Analysis Toolkit (GATK; v3.3-0). Variants with a quality score of >30 and an alignment quality score of >20 were annotated with SeattleSeq SNP Annotation (http://snp.gs.washington.edu/ SeattleSeqAnnotation141/). Rare variants present at a frequency of >1% in dbSNP 138 and the NHLBI GO Exome Sequencing Project or those present in local exomes of unaffected individuals were excluded. Variant prioritization focused on *de novo* heterozygous mutations and compound heterozygotes or hemizygotes affecting the coding sequence (missense, non-sense, and splice-site variants and coding indels). Candidate variants were inspected with the Integrative Genomics Viewer (https://www.broadinstitute.org/igv/) and confirmed by performing Sanger sequencing. Sequence variants were numbered starting from the first base of the ATG codon based on the numbering pattern of reference sequences. Variants were named using the Alamut 2.6.1 software (Interactive Biosoftware, Rouen, France) by following the Human Genome Variation Society nomenclature. The variation was searched across the Human Gene Mutation Database (http://www.hgmd.cf.ac.uk/ac/index.php), Genome Aggregation Database (http://gnomad.broadinstitute.org/about), NCBI Clinvar (https://www.ncbi.nlm.nih.gov/clinvar/) and Gene4Denovo (http://www.genemed.tech/gene4denovo/). SIFT, Polyphen-2, CADD, M-CAP, FATHMM, MutationTaster, Mutation Assessor and dbscSNV were used to predict mutations. On the basis of the results obtained from the abovementioned experiments, the pathogenicity of these mutations was determined in accordance with the American College of Medical Genetics (ACMG) clinical variant interpretation guidelines.

## Results

### Clinical Profiles

All the seven patients were born to unrelated Han Chinese parents through normal pregnancies.

Patient 1 is a 5-year-old girl, who was observed to have severe hypotonia in her infancy and developmental delay. She was incapable of lifting her head when she was 7-month-old as well as was incapable of sitting by herself and had trouble imitating speech or actions when she was 1-year-old. By the age of 14 months, she was found to lack the ability to maintain eye contact and displayed head-shaking, hand-clapping and hand-wringing as well as bruxism. While she exhibited purposeful hand use, she presented recurrent, febrile or non-febrile, generalized tonic-clonic seizures at the age of 18 months. After receiving treatment with Levetiracetam (LEV) at 20 months, she achieved seizure-free status at the age of 2 years. At the age of 18 months and 2 years, the findings of her EEG tests revealed spike-and-slow waves with bilateral temporal involvement. Her MRI findings at the age of 14 months indicated a widened subarachnoid space (Table 1).

**Table 1:** Clinical phenotype of patients with MEF2C gene mutation.

**No**.	**P1**	**P2**	**P3**	**P4**	**P5**	**P6**	**P7**
**Age**	**5 years**	**5 years 9 months**	**4 years 4 months**	**2 years 10 months**	**6 years**	**2 years 7 months**	**2 years 5 months**
Sex	F	F	F	F	F	M	F
Genetic defect	c.9A>T, (p.Arg3Ser)	c.78delT, (p.Phe26Leufs*3)	c.833_834insTT, (p.Leu278Phefs*1)	c.55-2(1VS2)A>G	C.44G>C, (p.R15P)	c.1A>G, (p.Met1Val)	c.3G>A, (p.Met1?)
Origin	Unknown	*De novo*	*De novo*	*De novo*	*De novo*	*De novo*	Unknown
Variation type	Missense	Frameshift	Frameshift	Splice site	Missense	Affecting the translation initiation site	Affecting the translation initiation site
Febrile seizure	+	+	+	+	-	+	-
Seizure type	Recurrent FS, GTC	Recurrent FS, GTC	Recurrent FS, absence	Recurrent FS, dystonic	GTC, IS	Recurrent FS, GTC	-
Age of seizure onset (months)	1.5 years	10 months	8 months	11 months	6 months	9 months	NA
ASM	LEV	LEV, OXC	LEV, VPA	OXC	VGB, LEV, VPA	VPA, OXC, TPM	NA
ASM response	Seizure free	Seizure free	Seizure free	Seizure free	Seizure free	No response	NA
Stereotypic movements	+	+	+	+	-	-	+
Poor hand skills	-	-	-	-	+	-	+
Delays in milestones	+	+	+	+	+	+	+
Independent sitting/age	1 year	1 year	8–9 months	1 year	-	9 months but regressed	2 years
Independent walking/age (months)	-	2 years	2 years	-	-	-	-
Infantile dystonia	Hypotonia	-	Hypotonia	Hypotonia	Hypotonia	Hypotonia	Hypotonia
Speech	-	-	-	-	-	-	-
Regression	-	+	-	+	-	+	-
Poor eye contact/autistic features	+	-	+	-	+	-	+
MRI	Frontal subarachnoid space enlargement	Normal	Delayed myelination	Normal	Delayed myelination	Small splenium of callosum	Normal
EEG	Bilateral temporal spike-slow waves	Bilateral temporal and occipital spike-slow waves	Generalized sharp-slow waves	Normal	Hypsarrhythmia	Left temporal and occipital slow waves	Left occipital 2–3 Hz sharp-slow waves in sleep

*FS, febrile seizure; GS, generalized seizures; GTC, generalized tonic-clonic seizures; IS, infantile spasms; LEV, levetiracetam; NA, not available; OXC, oxcarbazepine; PB, phenobarbital; PS, partial seizure; TPM, topiramate; VGB, vigabatrin; VPA, valproic acid; WMH, white matter hyperintensity*.

Patient 2 is a female who is currently 5 years 9 months old. She exhibited a delay in achieving some developmental milestones, such as head lifting at 4 months, independent sitting at 1 year and independent walking at 2 years. She could say “baba,” or “mama” at the age of 1 year but exhibited regression in development with subsequent instances of only unconscious articulation. Nevertheless, she possessed non-verbal communication skills. Hand clapping was observed at 8 months. She displayed good eye contact as well as a purposeful hand use. She presented recurrent, febrile or non-febrile, generalized tonic-clonic seizures at 10 months; after being treated with LEV at the age of 15 months, she achieved a seizure-free status. Her seizure, which was characterized as a generalized tonic seizure without fever, relapsed at the age of 3 years and was treated with valproate (VPA) and LEV. She achieved a seizure-free status when she was 3.5 years old. EEG (15 months) findings revealed spike-and-slow waves with bilateral temporal and occipital involvement. MRI findings at the age of 10 months were normal (Table 1).

Patient 3 is a female who is currently 4 years 4 months old. She showed some signs of developmental delay, such as lifting her head at the age of 5 months, sitting independently at the age of 9 months, and walking at the age of 2 years; she also did not exhibit any non-verbal communication skills and could not imitate actions or babble. Poor eye contact was observed at the age of 8 months. Hand clapping and hand wringing were observed when she was 1 year 6 months old. She demonstrated purposeful hand use, and at the age of 8 months, she experienced recurrent, febrile or non-febrile, generalized tonic-clonic seizures. She presented with status epilepticus, which later became absence status epilepticus at the age of 3 years 2 months. At this time, she was started on LEV medication but showed no response to it; subsequently, VPA was added to the treatment strategy at the age of 3 years 6 months, and the patient finally achieved a seizure-free status at the age of 4 years. Her EEG tests at the age of 15 months demonstrated sharp-slow synthetical waves at the left central and left parietal-frontal regions. EEG showed generalized sharp-slow waves and MRI findings indicated delayed myelination in the periventricular area at the age of 3 years 2 months (Table 1).

Patient 4 is a female who is currently 2 years 10 months old. Developmental delay was observed with regard to lifting of her head only at the age of 7 months and the ability to sit independently only at the age of 1 year; she also did not have the ability to walk independently and did not display any non-verbal communication skills. Although she could say the words “baba” or “mama” at the age of 2 years, she displayed a regression in development and only showed signs of unconscious articulation subsequently. Poor eye contact, head shaking, hand clapping, hand wringing, and bruxism were observed at the age of 1 year 3 months. Purposeful hand use was presented and retained. She experienced recurrent, febrile or non-febrile, generalized tonic-clonic seizures at the age of 11 months. Dystonic seizure occurred at 14 months. LEV was administered at the age of 16 months and the patient achieved seizure-free status at the age of 2.5 years. Her EEG (15 months and 2.5 years) and MRI (3 year) findings revealed normal findings (Table 1).

Patient 5 is a female who is currently aged 6 years; she was observed to have severe hypotonia at infancy and exhibited developmental delay. She could lift her head at 4 months but has not been able to sit or walk independently; she also did not display any non-verbal communication skills and was unable to imitate or babble. Poor eye contact was observed at 5 months. She did not exhibit purposeful hand usage. She presented generalized tonic-clonic seizure at the age of 6 months, which was treated with VPA; the patient responded positively to this treatment at the age of 7 months. However, seizure, which was characterized as spasms and of the tonic-clonic type, recurred at 1 year and 7 months; adrenocorticotropic hormone was used to treat this did not produce a positive outcome. Subsequently, vigabatrin, LEV and VPA were used for treatment; this treatment had a positive effect, and the patient achieved seizure-free status at the age of 2 years 7 months. Her EEG test revealed spike-slow waves with bilateral occipital involvement at the age of 6 months and hypsarrhythmia at 2 years of age. MRI scans taken at the age of 2 years showed delayed myelination in the periventricular region (Table 1).

Patient 6 is a male who is currently 2 years 7 months old. He was observed to have severe hypotonia in his infancy and developmental delay. He was incapable of lifting his head at the age of 5 months but could sit independently at 9 months; however, he exhibited a regression in development and was unable to walk independently. He also did not show any non-verbal communication skills and was unable to imitate or babble. Poor eye contact, head shaking, hand clapping and hand wringing were observed at the age of 1 year and 6 months. The patient displayed purposeful hand usage. He also presented recurrent, febrile or non-febrile, generalized tonic-clonic seizures at the age of 9 months. Topiramate, VPA and LEV were used but none of them showed a positive effect; this treatment was withdrawn at the age of 2 years. At present, the patient's daily seizures have been characterized as myoclonic. EEG findings obtained at the age of 10 months revealed spike-slow waves with bilateral-central-frontal involvement. MRI scans taken at the age of 15 months showed developmental malformation of the corpus callosum (Table 1).

Patient 7 is a female who is currently 2 years 5 months old. She experienced severe hypotonia at infancy and was observed to have developmental delay. She was incapable of both lifting her head at the age of 7 months and sitting independently at the age of 2 years; she was also unable to walk independently, demonstrated no non-verbal communication skills, and was unable to imitate or babble. Poor eye contact as well as head-shaking and hand-clapping behavior were observed at the age of 1 year. She did not display purposeful hand usage. She did not experience seizure. EEG test conducted at the age of 2 years revealed 2–3 Hz sharp-slow waves at the left parietal-occipital region. MRI scans taken at the age of 14 months showed malformation of the corpus callosum, delayed myelination and leukomalacia in the periventricular region (Table 1).

#### Sequencing Analysis of Trio-WES Tests

Trio-WES revealed two ambiguously inherited (P1, c.9A>T, p.Arg3Ser, P1's mother, c.9A>T, mosaic, 10/119; P7, c.3G>A, p.Met1, P7's mother, c.9A>T, mosaic, 8/113) and five *de novo* [P2, c.78delT, p.Phe26Leufs^*^3; P3, c.833_834insTT, p.Leu278 Phefs^*^1; P4, c.55-2 (1VS2) A>G; P5, c.44G>C, p.Arg15Pro; P6, c.1A>G, p.Met1Val] heterozygous mutations in the MEF2C gene. All these mutations were searched across the databases mentioned in the section Methods; because P2 and P3 mutations lead to the formation of a truncated protein, the other five mutations were assessed using the mutation prediction tools mentioned previously. Subsequently, the clinical implications and considerations for evaluating *in silico* algorithms that can be used along with the clinical variant interpretation guidelines established by the American College of Medical Genetics were investigated; five mutations were classified as pathogenetic, two mutations were considered to be likely pathogenic (more details see Supplementary Table 1).

## Discussion

In cases where the gene is abnormally expressed owing to mutation or deletion and a lower level of protein is produced; therefore, insufficient material for maintaining normal cellular physiological functions leads to MCHS (1). Patients with MCHS often exhibit delayed developmental milestones, severe intellectual disabilities, limited or absent speech, seizures, hypotonia, limited walking abilities, and stereotypic movements (12).

All seven patients in our cohort presented delays in developmental milestones, severe intellectual disabilities, and absent speech. Six patients exhibited infantile hypotonia and five cases showed stereotypic movements; these symptoms were also common in the 23 patients reported previously (100, 100, 95.7, 78.2, and 73.9%) (1, 6–14). Four cases in our cohort had poor eye contact, indicative of autism, while two cases showed poor hand skills. Of the 23 patients in the previous study (1, 6–14), 18 were evaluated for autism-related manifestations and 13 of these patients reported autism-like manifestation; furthermore, 14 of the 23 patients were evaluated for hand function of whom 5 were reported to have poor hand skills. The results of our study are consistent with the proportion of these two manifestations reported in previous studies. Brain MRI scans revealed abnormalities in five cases, similar to those reported previously (12); however, the manifestations were varied and included delayed myelination, frontal subarachnoid space enlargement, and corpus callosum dysgenesis. Wang et al. (13) noted that MCHS was more common in females than in males, but the same pattern was observed in our cohort. It is worth noting that in our study, five patients were unable to work, which is significantly higher than the proportion reported in the literature. However, at present, we cannot comment on whether the children in our cohort may acquire the ability to work in the future.

Six patients experienced seizure, including five with onset at infancy, which was consistent with the proportion reported in previous studies; 18 patients of the 23 patients analyzed were found to experience seizures, and 12 of those patients showed onset when they were <1-year-old (8, 12). Febrile seizures were very common in our cohort (5/7); similarly, three out of five patients described by Wang et al. had febrile convulsions (13). Previous descriptions of seizure outcome in such patients are limited as only seven patients had described the response to antiseizure medicine (ASM). Six achieved seizure-free status, and one patient showed partial response (1, 8, 11, 13, 14). At present in our cohort, six patients received ASM and only one patient experienced refractory epilepsy. Seizures experienced by the other five patients were effectively controlled, including the case with infantile spasm, which is a severe form of epilepsy. Most patients with epilepsy and MCHS exhibited good responses to ASM and achieved a seizure-free status. There does not seem to be a significant difference between the choice of ASM and treatment outcome.

Regression has only been described in two patients with MCHS (1, 13). Contrary to the reports found in the literature, three patients (P2, P4, and P6) in our cohort also showed clinical regression. Zweier et al. concluded that the loss of MEF2C function in humans can significantly reduce the gene expressions levels of MeCP2 and CDKL5, and MEF2C mutations reduce synergistic reactivation of the E-box promoter of MeCP2 and CDKL5 (15). MeCP2 is the pathogenic gene responsible for Rett syndrome (16), and the stereotypical loss of motor and language skills and developmental delay encountered in autism were also observed in patients with Rett syndrome; this result overlaps with MCHS and causes some difficulties in MCHS diagnosis. However, delayed early life development may help distinguish between the two conditions.

Sixteen MEF2C point mutations were previously described (1, 7–9, 11–14). Seven patients in our cohort had MEF2C point mutations, including six novel mutations. Specifically, two variants (c.3G>A, p.Met1?; c.1A>G, p.Met1Val) affecting the translation initiation site had not been described in previous studies. On reviewing previous studies as well as our cohort, we found an interesting phenomenon: patients with intragenic deletions seemed more likely to be ambulatory than the patients with point mutations in MEF2C (6/7 vs. 9/23). This contradicts with the observations reported by Wang et al.; however, we need to investigate more cases to support our hypothesis. Variants affecting the translation initiation site have not yet been reported. Patients 6 and 7 in our study were affected by this type of point mutation, but they exhibited different clinical phenotypes. P6 showed persistent drug-resistant epilepsy and developmental regression, whereas P7 did not.

**Figure 1:**
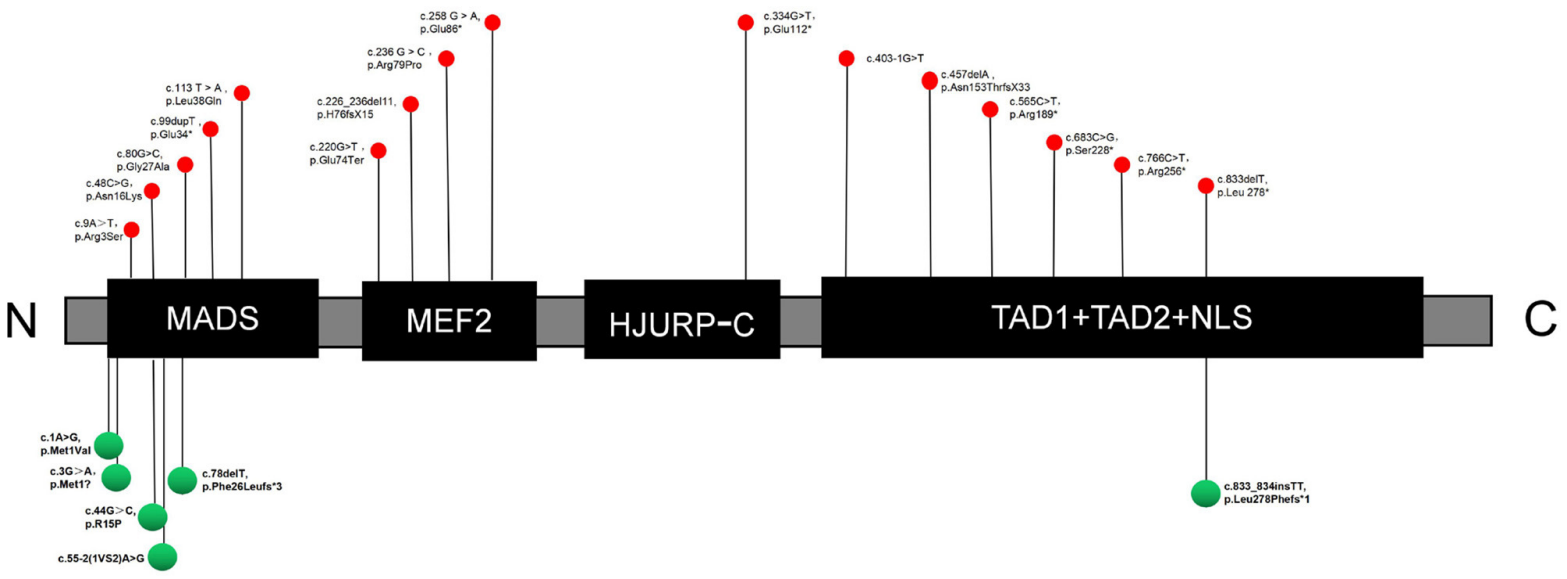
Sites of mutations in the MEF2C protein. Red and green indicate reported mutations and novel mutations detected in this study, respectively. C, C-terminus; N, N-terminus; MADS, MCM1, agamous, deficiens, serum response factor; MEF2, myocyte enhancer factor 2; HJURP-C, Holliday junction recognition protein C-terminal; NLS, nuclear location signal; TAD, transcriptional activation domain.

The human MEF2C protein consists of six core structural domains, including MADS (MCM1, agamous, deficiens, serum response factor), myocyte enhancer factor 2 (MEF2), HJURP-C (Holliday junction recognition protein C-terminal), transcriptional activation domain 1 (TAD1), transcriptional activation domain 2 (TAD2) and a nuclear localization signal (NLS). The MADS domain contains 56 amino acids, while the MEF2 domain spans amino acids 57–86. The HJURP-C domain consisting of 29 amino acids was adjacent to the MEF2 domain and followed by two transcriptional activation domains (TAD1 and TAD2), which are responsible for activating transcription. The NLS domain (nuclear localization signal) is located at the C-terminus of MEF2C, which controls nuclear translocation of the protein. The MADS and MEF2C domains mediate dimerization and DNA binding, and the other domains act as transcriptional activators (17). The reported mutation sites (including those in our study) do not have a specific distribution within the structural domains, and there are no obvious phenotypes indicating mutation sites in hot spots (Figure 1). Among our small cohort, there was no obvious correlation between clinical phenotype and mutation sites across different domains.

To date, only 30 patients with MEF2C point mutations, including the 7 cases described here, have been identified (1, 6, 7, 9, 11–14), which limited our ability to analyze correlations. There was no obvious genotype–phenotype correlation after classification, suggesting that more clinical cases are needed for conducting further analyses. All mutations reported in the published papers were *de novo*, and two of the seven patients (P1 and P7) in our cohort had mutations whose inheritance pattern remains unclear, with their mothers having low-level mosaicism (mother of P1: 10/119; mother of P7: 8/113). MCHS was previously considered to be an autosomal dominant disease, and healthy mosaicism has not yet been reported. To our knowledge, this is the first description of such a phenomenon, which leads us to speculate that a low-level mosaicism does not cause MCHS.

This was a small cohort study, so we cannot draw firm conclusions about the phenotypic–genotypic associations. Similarly, owing to technical reasons and the subjects' refusal to undergo further studies, we could not further analyze the genetic patterns of the two cases; however, we suspect that the patients inherited the mutated gene from the mother in both instances as their mothers carried the same mutation.

## Conclusion

We described six novel MEF2C gene mutations. Although the clinical manifestations of MCHS are diverse, common features include global developmental delay, impaired language function, seizure, stereotyped behavior, infantile hypothermia, and abnormal brain MRI findings. Although regression was not a common phenomenon, it was reported to occur occasionally. Currently, it has been difficult to establish a clear genotype-phenotype relationship in MCHS, which should be the focus of future research. Although patients with MCHS often experience seizures during the early stages of their life, most patients eventually achieve a seizure-free status. People without any MCHS symptoms may also have low-level mosaicism and carry the pathogenic MEF2C mutation that can be passed on to their offspring.

## Data Availability Statement

The data presented in the study are deposited in the China National Genebank repository, accession number is CNP0001827.

## Ethics Statement

The studies involving human participants were reviewed and approved by Ethics Committee of the PLA General Hospital. Written informed consent to participate in this study was provided by the participants' legal guardian/next of kin.

## Author Contributions

LW and GY wrote the first draft of this manuscript. LZ and GY contributed to study conception and design. All authors helped revise the manuscript with regard to important intellectual content.

## Conflict of Interest

The authors declare that the research was conducted in the absence of any commercial or financial relationships that could be construed as a potential conflict of interest.

## Abbreviations

EEG: electroencephalogram
MCHS: MEF2C haploinsufficiency syndrome
MRI: magnetic resonance imaging
Trio-WES: trio-based whole-exome sequencing.

## References

[1] Le Meur N Holder-Espinasse M Jaillard S Goldenberg A Joriot S Amati-Bonneau P. MEF2C haploinsufficiency caused by either microdeletion of the 5q14.3 region or mutation is responsible for severe mental retardation with stereotypic movements, epilepsy and/or cerebral malformations. J Med Genet. (2010) 47:22–9. 10.1136/jmg.2009.069732 19592390 PMC2848840

[2] Novara F Beri S Giorda R Ortibus E Nageshappa S Darra F. Refining the phenotype associated with MEF2C haploinsufficiency. Clin Genet. (2010) 78:471–7. 10.1111/j.1399-0004.2010.01413.x 20412115

[3] Shalizi A Gaudillière B Yuan Z Stegmüller J Shirogane T Ge Q. A calcium-regulated MEF2 sumoylation switch controls postsynaptic differentiation. Science. (2006) 311:1012–7. 10.1126/science.1122513 16484498

[4] Barbosa AC Kim MS Ertunc M Adachi M Nelson ED McAnally J. MEF2C, a transcription factor that facilitates learning and memory by negative regulation of synapse numbers and function. Proc Natl Acad Sci USA. (2008) 105:9391–6. 10.1073/pnas.0802679105 18599438 PMC2453723

[5] Li Z McKercher SR Cui J Nie Z Soussou W Roberts AJ. Myocyte enhancer factor 2C as a neurogenic and antiapoptotic transcription factor in murine embryonic stem cells. J Neurosci. (2008) 28:6557–68. 10.1523/JNEUROSCI.0134-08.2008 18579729 PMC2679693

[6] Srivastava S Cohen JS Vernon H Barañano K McClellan R Jamal L. Clinical whole exome sequencing in child neurology practice. Ann Neurol. (2014) 76:473–83. 10.1002/ana.24251 25131622

[7] Zweier M Gregor A Zweier C Engels H Sticht H Wohlleber E. Mutations in MEF2C from the 5q14.3q15 microdeletion syndrome region are a frequent cause of severe mental retardation and diminish MECP2 and CDKL5 expression. Hum Mutat. (2010) 31:722–33. 10.1002/humu.21253 20513142

[8] Bienvenu T Diebold B Chelly J Isidor B. Refining the phenotype associated with MEF2C point mutations. Neurogenetics. (2013) 14:71–5. 10.1007/s10048-012-0344-7 23001426

[9] Paciorkowski AR Traylor RN Rosenfeld JA Hoover JM Harris CJ Winter S. MEF2C Haploinsufficiency features consistent hyperkinesis, variable epilepsy, and has a role in dorsal and ventral neuronal developmental pathways. Neurogenetics. (2013) 14:99–111. 10.1007/s10048-013-0356-y 23389741 PMC3773516

[10] Tanteles GA Alexandrou A Evangelidou P Gavatha M Anastasiadou V Sismani C. Partial MEF2C deletion in a Cypriot patient with severe intellectual disability and a jugular fossa malformation: review of the literature. Am J Med Genet A. (2015) 167A:664–9. 10.1002/ajmg.a.36945 25691421

[11] Rocha H Sampaio M Rocha R Fernandes S Leão M. MEF2C haploinsufficiency syndrome: report of a new MEF2C mutation and review. Eur J Med Genet. (2016) 59:478–82. 10.1016/j.ejmg.2016.05.017 27255693

[12] Vrečar I Innes J Jones EA Kingston H Reardon W Kerr B. Further clinical delineation of the MEF2C haploinsufficiency syndrome: report on new cases and literature review of severe neurodevelopmental disorders presenting with seizures, absent speech, and involuntary movements. J Pediatr Genet. (2017) 6:129–41. 10.1055/s-0037-1601335 28794905 PMC5548525

[13] Wang J Zhang Q Chen Y Yu S Wu X Bao X. Novel MEF2C point mutations in Chinese patients with Rett (-like) syndrome or non-syndromic intellectual disability: insights into genotype-phenotype correlation. BMC Med Genet. (2018) 19:191. 10.1186/s12881-018-0699-1 30376817 PMC6208086

[14] Borlot F Whitney R Cohn RD Weiss SK. MEF2C-related epilepsy: delineating the phenotypic spectrum from a novel mutation and literature review. Seizure. (2019) 67:86–90. 10.1016/j.seizure.2019.03.015 30922778

[15] Zweier M Rauch A. The MEF2C-related and 5q14.3q15 microdeletion syndrome. Mol Syndromol. (2012) 2:164–70. 10.1159/000337496 22670137 PMC3366707

[16] Sandweiss AJ Brandt VL Zoghbi HY. Advances in understanding of Rett syndrome and MECP2 duplication syndrome: prospects for future therapies. Lancet Neurol. (2020) 19:689–98. 10.1016/S1474-4422(20)30217-9 32702338

[17] Dong C Yang XZ Zhang CY Liu YY Zhou RB Cheng QD. Myocyte enhancer factor 2C and its directly-interacting proteins: a review. Prog Biophys Mol Biol. (2017) 126:22–30. 10.1016/j.pbiomolbio.2017.02.002 28163053

